# Co‐Designing a Sensory Support Intervention for Older Adults With Hearing and Vision Impairment Living in Australian Home Care Settings

**DOI:** 10.1111/hex.70481

**Published:** 2025-11-06

**Authors:** Helen Gurteen, Melinda Toomey, Bronwyn Franco, Najwan El‐Saifi, Chyrisse Heine, Iracema Leroi, Carly J. Meyer, Sheela Kumaran, Piers Dawes

**Affiliations:** ^1^ Centre for Hearing Research, School of Health and Rehabilitation Sciences University of Queensland Brisbane Australia; ^2^ Institute of Health and Wellbeing Federation University Australia Ballarat Australia; ^3^ Global Brain Health Institute, Trinity College Dublin The University of Dublin Dublin Ireland; ^4^ Bolton Clarke Research Institute Brisbane Australia; ^5^ School of Optometry and Vision Science UNSW Sydney Sydney Australia

**Keywords:** aged care, co‐design, complex intervention, hearing impairment, vision impairment

## Abstract

**Background:**

Home care services help older adults remain living independently in their own homes. Approximately 70% of home care recipients have hearing and/or vision impairments, and 20% likely have cognitive impairments. A home‐based hearing and vision intervention (SENSE‐Cog), incorporating impairment identification and correction, device and communication training, goal setting, referrals, provision of supplementary devices, and fostering social inclusion, was originally co‐developed with people with dementia in Europe.

**Aim:**

We aimed to co‐design an adaptation of the intervention for older Australians in home care settings.

**Methods:**

An iterative co‐design process with audiologists, eye care professionals, home carers and research experts identified modifications required for the intervention. Themes from workshops guided modifications, and feedback from research experts and three public and patient involvement workshops provided additional usability insights.

**Results:**

Thematic analysis indicated the potential benefits of the intervention related to establishing a strong foundation, decision support, timely access to care and effective communication. Challenge‐related themes centred around resistance and denial, cultural and linguistic barriers, cognition, age, implementation, and sensory therapist‐related challenges. Modification themes closely paralleled the challenges of incorporating topics such as tailored support, information provision, extended training, addressing disparities and sensory therapist development.

**Discussion:**

The co‐design process led to modifications of intervention components to meet the needs of the Australian population and the creation of a Sensory Therapist training module. A field trial is planned to assess the efficacy of the adapted intervention.

**Patient or Public Contribution:**

Older adults with lived experience caring for a family member with dementia played a central role in reviewing the developed intervention materials as part of PPI workshops. They provided unique insights into the usability of the materials, and these were used to refine the intervention to better suit the older adult population. Key differences existed between the insights offered by the public and by research experts, highlighting the value of public contributions to intervention development.

## 1 Introduction

Home care services are a pivotal component of the Australian aged care sector. These services are government‐subsidised programs that support older adults to live independently in their own homes, reducing demand for residential aged care [1]. Remaining in one's own home is a desirable outcome for older adults, with research showing that the entry into residential aged care is associated with feelings of grief and stress [2]. However, remaining at home brings its own challenges as it may heighten loneliness and social isolation [3]. The Royal Commission into Aged Care Quality and Safety highlights the need for integrated, community‐connected care that allows older adults to continue living fulfilling lives at home [1].

Within the Australian home care population, over 70% of recipients experience hearing and/or vision impairment [4, 5]. The high percentage of people experiencing vision and hearing impairment is unsurprising given the well‐demonstrated correlation between these conditions and increasing age. A population survey by Wilson et al. found that 62.8% of Australian adults over the age of 70 years have hearing loss (> 25 dB HL in the better ear) [4]. The prevalence of vision loss in Australians over the age of 65 years is 6.7% (defined as corrected better eye visual acuity less than 6/12) [6]. Schneider et al. found that over 26% of community‐dwelling Australian adults over the age of 80 years experience dual sensory loss (defined as visual acuity less than 6/12 in the better eye and pure‐tone air conduction threshold > 25 dB HL in the better ear) [7].

In comparison, the National Health and Aging Trends Study found that 65.3% of community‐dwelling older adults in the United States over the age of 71 years have hearing loss (> 25 dB HL in the better ear) [8]. Within the same sample, 27.8% had vision loss (defined as corrected better ear visual acuity worse than 20/40) [9] and 22% experienced dual sensory loss [10]. The high levels of hearing, vision and dual sensory loss within older adults suggest that sensory impairments are a significant public health issue. Untreated sensory impairment has been shown to be associated with poorer mental health, social isolation, reduced physical activity and reduced quality of life [11].

Because both sensory and cognitive impairments are strongly associated with age, hearing and vision impairment is commonly comorbid with cognitive impairment. Cognitive impairment can be defined as a measurable deficit in cognitive domains such as memory, attention or executive function [12]. A longitudinal study of adults over 60 years of age highlighted that worse visual acuity correlates with greater declines in cognitive performance over time [12]. A similar survey reported 1.9‐ to 2.8‐fold higher odds of cognitive impairment amongst those with visual impairment (defined as worse than 20/40 vision) compared to those with good vision [13]. Similarly, older adults with moderate to severe hearing loss were reported to be 1.61 times more likely to have dementia, which involves a substantial decline in cognitive function, than those with hearing within normal limits [14].

Hearing and vision impairments exacerbate the impact of cognitive impairment on quality of life, functional ability and social engagement [15]. Unfortunately, hearing and vision impairment is more likely to go unrecognised and untreated among people with cognitive impairment than in those with normal cognition [16]. This may be in part due to challenges surrounding the appropriate assessment of hearing and vision impairment for this population, or the challenges differentiating between difficulties caused by hearing and vision impairment and those attributable to cognitive impairment [17].

Intervening to address hearing and vision impairments has the potential to improve the quality of life for older adults. Hearing and vision interventions offer a low‐risk and relatively low‐cost non‐pharmacological intervention that is desired by patients [15]. A synthesis of the literature identified a range of hearing interventions utilising approaches such as hearing aid/cochlear implant fitting and training, communication training, and fitting of assistive listening devices [15]. In the same review, vision interventions were found to have incorporated fitting of prism lenses, vision rehabilitation training and cataract surgery [15]. While the quality of the evidence was found to be low to moderate overall, the review showed that vision and hearing interventions can improve sensory‐related outcomes for people living with cognitive impairment or dementia, but that significant challenges exist in maintaining hearing aid use and that existing intervention studies have not been adequately individualised to patterns of impairment [15, 18]. The use of hearing and vision interventions, such as those discussed by Dawes et al. [15], is further complicated by the presence of dual sensory loss, where compensation for a loss in one sense by the other is not possible. A systematic review of care practices for individuals with dual sensory loss identified a need for targeted intervention appropriate for this population's needs [19]. These challenges point to a need to consider interventions that expand support for hearing and vision needs beyond traditional care pathways, which are often limited to device provision with limited informational counselling, especially for those living with dementia or cognitive impairment.

A sensory support intervention, SENSE‐Cog, was co‐developed in Europe with people living with dementia to address the hearing and/or vision care needs of older adults with dementia [20]. The intervention is a complex, multidimensional, individualised intervention, delivered over a 12‐week period, which aims to improve outcomes related to quality of life, behaviour and mood, functional ability, and social connectedness for older adults with comorbid sensory and cognitive conditions, as well as reducing burden and improving relationship quality for their informal caregivers [21]. This is achieved by extending the support period past the fitting of spectacles and hearing aids, to enhance the likelihood that improvements are long‐lasting [21].

The intervention was developed using the COM‐B (Capability, Opportunity, Motivation, Behaviour) model [22], an evidence‐based model of behaviour change. Each component of the intervention addresses capability, opportunity or motivation to promote the desired behavioural outcomes (e.g., sensory device use) [21]. The seven components of the intervention are delivered by an allied health professional such as an occupational therapist or psychologist, referred to as a sensory therapist, and include identification and correction of any vision or hearing impairment, continuous training in the correct use of sensory devices, communication training, home‐based functional assessment and goal setting, referral to health and social services, provision of supplementary sensory devices, and fostering social inclusion through hobbies/interests/social groups [21].

A field trial of the SENSE‐Cog intervention was conducted with 19 older adults who had hearing and/or vision loss and a formal diagnosis of mild to moderate dementia and their care partners [23]. The intervention was found to be feasible, tolerable and acceptable to the intervention recipients, with clinically significant improvements in quality of life [24]. A full‐scale randomised control trial reported short‐term improvements in quality of life, but these were not maintained in the long term [25]. The trial was impacted by the Covid‐19 pandemic, which prevented implementation of the intervention in its originally intended form [25]. The study experienced delays in device fitting, an inability to deliver face‐to‐face interventions as planned, and a reduced number of appointments per participant [25]. Each of these alterations to the intervention may have impacted the study outcomes.

Given the prevalence of hearing, vision and cognitive impairments in the Australian home care setting, and the lack of an intervention developed with this setting in mind, our goal was to adapt and evaluate the European SENSE‐Cog intervention for Australian home care service users, including those living with dementia. Skivington et al. highlighted that the context in which an intervention is delivered may impact the outcome of the intervention itself [26]. Key differences exist between the Australian and European contexts in the provision of hearing and vision services. For example, the provision of vision care varies on a state‐by‐state basis in Australia, with different levels of access and support for eye health services [27]. Further, Australia utilises a blended system for hearing care provision, with government‐funded care available to a subset of the population dependent on age and means testing, with non‐eligible individuals (e.g., self‐funded retirees) required to fund their own devices through either private health insurance or out of pocket [28]. Across Europe, models of hearing and vision care vary between countries, with countries such as the United Kingdom funding hearing aids for all, while countries such as Belgium and Italy have very limited access to hearing aid provision and funding [29].

The current study aimed to work with older adults and subject matter experts to co‐adapt the SENSE‐Cog intervention for Australian home care settings. The research questions posed were: (1) to what extent is the existing SENSE‐Cog Sensory Support Intervention appropriate for the Australian context and (2) what modifications are required to optimise the SENSE‐Cog Sensory Support Intervention for Australian home care settings?

## 2 Methods

### 2.1 Study Design

The adaptation of the SENSE‐Cog intervention for the Australian home care settings consisted of two parts. Part 1 used semi‐structured interviews to explore the support care needs of people with hearing and/or vision impairment receiving home care services. Briefly, these interviews, which were conducted with older adults with hearing and/or vision impairment who had varying cognitive statuses, and their informal caregivers, revealed unmet needs within the themes: (1) Understanding the individual is fundamental to personalised hearing and vision care, (2) Importance of support networks, (3) Enhancing device use and communication and (4) Home life and community. Further information regarding these needs was provided in Toomey et al. 2025 [30].

This paper reports the findings from part 2, which used the participatory design method of co‐design. Ethics approval was obtained from the University of Queensland Research Ethics Committee—approval number 2023/HE001081.

### 2.2 Co‐Design

Co‐design is a collaborative and inclusive approach that brings together diverse stakeholders, including researchers, practitioners and end users, to collectively design and shape interventions that address specific challenges or needs. Unlike traditional top‐down approaches, co‐design empowers participants by recognising their expertise and lived experiences as valuable contributions to the design process [31]. Through open dialogue, ideation and iteration, co‐design fosters a shared understanding of complex issues, leading to innovative solutions that are contextually relevant and user‐centric [31]. The co‐design evaluation framework highlights that consumer involvement in the co‐design process can be incorporated at multiple points from priority setting through to dissemination [32]. Numerous overlapping approaches to co‐design have been utilised in the literature with common activities including workshops, interviews and consumer advisory councils [33].

In the context of the current study, co‐design served as a dynamic framework to collaboratively adapt the existing evidence‐based SENSE‐Cog intervention to the unique requirements of the Australian context, ensuring a tailored solution for the identified support care needs of older Australians with hearing and/or vision impairments. Boyd et al.'s co‐design model was adopted for use in the current study due to its demonstrated effectiveness in healthcare settings [31].

### 2.3 Participants, Sampling and Inclusion

**Figure 1:**
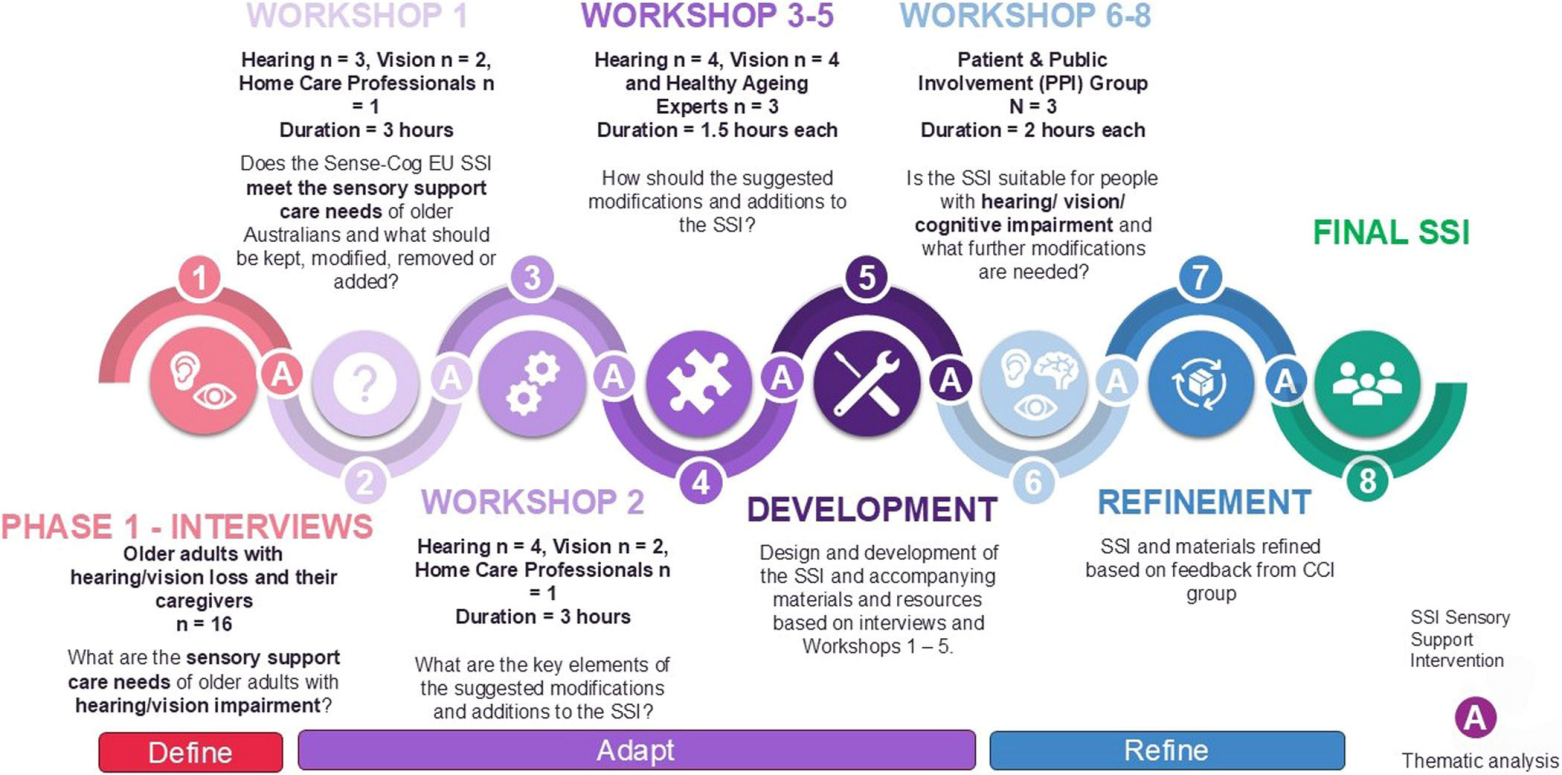
Co‐design elements mapped to indicate where adaptation and refinement of the intervention occurred. SSI = Sensory Support Intervention.

Participants in the co‐design included audiologists (*n* = 5), vision care professionals (*n* = 2), a paid home care worker employed by a community aged care provider (*n* = 1), healthy ageing researchers (*n* = 3) and hearing and vision researchers (*n* = 8). Figure 1 outlines the stages of the co‐design process where each participant group was involved. Participants were recruited via purposive sampling, identified through home care provider partner organisations, professional health associations, professional networks and professional interest groups as professionals with an interest in hearing, vision, dementia or aged care.

Audiologists and vision care professionals were eligible to participate if they worked clinically in Australia and provided care to older adults with hearing or vision impairment, respectively. Home care staff were eligible to participate if they worked for an Australian home care provider. Furthermore, all participants had to be able to consent and have conversational English [34]. Written informed consent was obtained from all participants.

### 2.4 Format of Co‐Design

An overview of the co‐design process is provided in Figure 1. It maps the participant groups involved in each stage and the key questions addressed. Availability of the hearing, vision and home care professional participants meant that there were differing numbers of participants for Workshops 1 and 2. The co‐design process was designed to be iterative, with input from each stage shaping the direction of the next. The entire process was conducted over a 12‐month period.

### 2.5 Workshops 1 and 2

The initial two virtual (Zoom, Zoom Communications, San Jose, California, the United States) workshops were held 2 weeks apart and used elements of ‘*engage*’, ‘*plan*’, ‘*explore*’, ‘*develop*’ and ‘*decide*’ from Boyd et al.'s co‐design model [35]. Workshop 1 commenced with introductions and icebreaker activities to facilitate participant interactions (*engage*). Participants were advised of the workshop's goal and vision, which was to modify the SENSE‐Cog European intervention to the Australian home care context (*plan*). The sensory support care needs of older Australians, identified in part one of the research [30], were shared to provide context. Brief didactic sessions and interactive activities on Padlet (Padlet, San Francisco, California, the United States) were used throughout Workshop 1 to facilitate the co‐design process (*explore, develop*) to adapt and modify the intervention. This included: seeking feedback regarding likes, dislikes and ideas using the Feedback Capture Grid [36], mapping support care need themes and sub‐themes to the intervention to identify unmet needs, deciding if the components should be kept, modified or removed to address unmet needs.

Workshop 2 further developed key areas of adaptation identified from Workshop 1 (*explore, develop*) and then rated the modified intervention using the APEASE criteria (Affordability, Practicality, Effectiveness & cost‐effectiveness, Acceptability, Side‐effects & safety and Equity) [37]. In addition, potential barriers and enablers to successful implementation of the modified intervention were explored using the Context and Implementation of Complex Intervention (CICI) framework (*explore*) [38]. The CICI framework assists in structural analysis of implementation context at micro (e.g., individual), meso (e.g., community or organisation) and macro levels (e.g., regional or national) across the dimensions of context (7 domains), implementation (5 domains) and setting.

### 2.6 Workshops 3–5

Workshops 3, 4 and 5 continued the development of the areas identified as in need of modification in Workshop 1 and fleshed out in Workshop 2. The various researchers were presented with a summary of the results of Workshops 1 and 2 (*engage*), and the scope of the co‐design process was outlined (*plan*). Short interactive engagement exercises were utilised to facilitate the exchange of ideas via Padlet and live discussion (*explore, develop*).

### 2.7 Analysis of Workshop Data

Preliminary data analysis was undertaken following Workshops 1 and 2 to facilitate deeper discussion of emerging themes. De‐identified transcripts were examined using inductive analysis to summarise common themes, following Braun and Clarke's method: data familiarisation, coding, theme identification, theme development, theme definition and theme verification [39].

For the preliminary data analysis, one author (M.T., optometrist) analysed the transcripts, with independent review by another author (H.G., audiologist) to ensure coding reliability. After the co‐design workshops, themes were refined further through collaborative discussions with three team members with expertise in audiology and optometry (B.F.).

### 2.8 Reflexivity Statement

This section reflects on how the experience and positionality of the authors have shaped the co‐design process. The workshops were facilitated by two core members of the research team (M.T. and H.G.), who brought distinct clinical and research backgrounds. M.T. is an optometrist with qualitative research experience, while H.G. is an audiologist with expertise in service design and implementation. Both have worked extensively with older adults, which informed their empathetic engagement with participants. Their professional identities and interpersonal dynamics influenced facilitation styles and the interpretation of participant input. To ensure rigour, a third team member (B.F.), also a clinical audiologist, contributed to thematic analysis. Throughout the co‐design process, we actively employed reflexivity strategies such as team debriefing discussions, individual reflections and tracking meeting notes.

### 2.9 Development of Intervention Materials and Usability Review

Two members of the research team (M.T. and H.G.) used the themes identified to guide the adaptation of the intervention materials (see Appendix A for an outline of each component within the intervention and materials developed). As with the original SENSE‐Cog Sensory Support Intervention, the materials were intended to be used throughout a 12‐week intervention period, facilitated by a sensory therapist. The adapted resources were developed within Canvas (Instructure, Salt Lake City, Utah, the United States), a web‐based learning management system (LMS) widely used by educational institutions. Canvas provides a robust platform for managing digital learning, allowing developers to create online course materials and facilitate communication and collaboration with users [40]. The intervention resources developed through the workshops were integrated into Canvas to leverage its customisable course creation tools and user‐friendly interface and to ensure an effective and engaging learning experience for all participants. Canvas provides several accessibility features which were leveraged to optimise hearing, vision and cognitive accessibility. Features used included: audio captioning of videos, high contrast mode, 14‐point font, alternate attributes on image elements, support for screen magnification, consistent structures to allow correct reading sequences to be programmatically determined, screen reader compatibility, no animations or advertisements to minimise distraction, step‐by‐step instructions, no blinking content, and large navigation buttons.

Researchers utilised Qualtrics (Qualtrics, Seattle, Washington State, the United States), an advanced online survey tool, to develop comprehensive questionnaires. These questionnaires are designed to capture detailed information about the personal sensory needs of intervention recipients, as perceived by the participants themselves, as well as gather insights from audiologists, optometrists and other health professionals involved in the project. This approach allows the sensory therapist to tailor interventions more effectively based on the specific needs and feedback of each participant. This individualisation has been shown to enhance the overall efficacy of interventions [15].

Care was taken to develop complementary paper‐based versions of all intervention resources. This decision was taken to ensure that any issues regarding computer literacy would not prevent intervention recipients from engaging with the materials and the sensory support program. Following initial development, the materials were shared via email with the researchers previously invited to participate in Workshops 3–5. They were provided with a period of 3 weeks in which they could peruse the materials and provide feedback to the research team via email regarding the suitability and usability of the research materials.

## 3 Results

**Figure 2:**
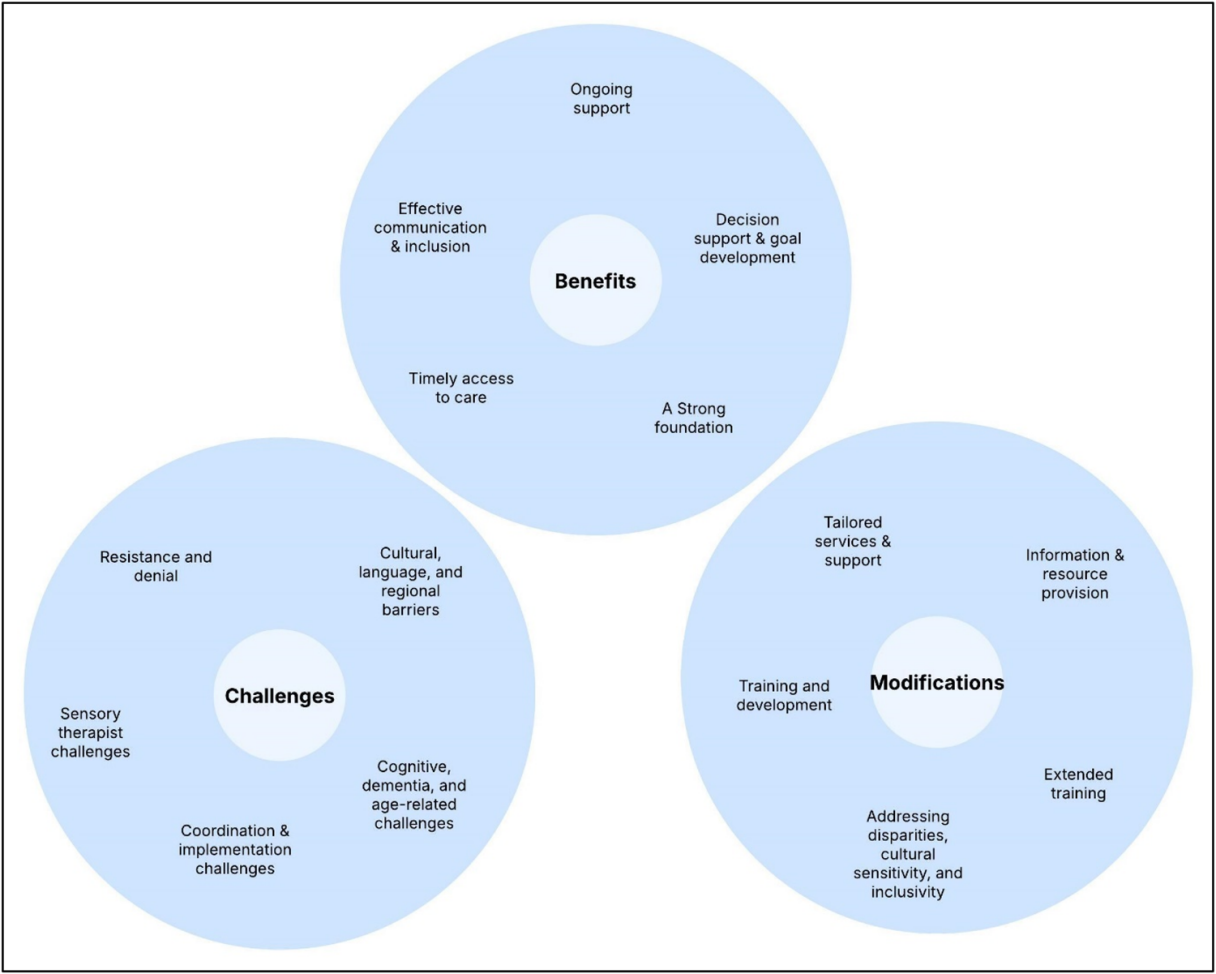
A visual representation of theme development.

A visual representation of the generated themes is provided in Figure 2. Themes were categorised into benefits, challenges and areas requiring modification. Participant quotes illustrating the final themes are presented in Table 1.

**Table 1:** A summary of all benefit, challenge and modification themes with illustrative quotes.

Benefit themes		Challenge themes		Modification themes	
Benefit theme summary	Representative quote	Challenge theme summary	Representative quote	Modification theme summary	Representative quote
**Benefit Theme 1: A strong foundation** This theme captures the advantages of having a sensory therapist to identify and address vision and hearing issues. This approach may alleviate pressure on family and friends while ensuring that home care recipients receive professional and objective recommendations for necessary checks.	‘*To have a trained sensory therapist to professionally identify vision and hearing issues and assist with referrals will take some of the load off family and friends. It also provides an objective and professional recommendation and push to have vision and hearing checks done’*.	**Challenge Theme 1: Resistance and denial** This theme includes resistance to intervention from individuals and denial of issues from families. It also covers perceived coping, where some home care recipients may feel they are coping well with their vision and hearing, potentially resisting intervention or assessments.	‘*Some people have the attitude of “don′t care” anymore. So it′s not easy to influence or change their mindset’*. ‘*Sometimes the family doesn′t want to admit their parent has an issue, so they tell them they will be fine, no other action is needed, and the person will not take action to avoid the problems’*.	**Modification Theme 1: Tailored services and support** This theme emphasises the importance of recognising and addressing the unique challenges faced by older adults, particularly those with hearing and vision loss or cognitive impairment. It highlights the necessity of customising programs across multiple dimensions: both in the approach taken during individual sessions and in the long‐term management of hearing and vision health. This includes monitoring and responding to changes in hearing or vision over time.	‘*Individualised support around device use for people with dual sensory loss, e.g., Rechargeable hearing aids for safety, tactile puff paint, use of Velcro on the device’*. ‘*… people may have trouble goal setting independently and may need caregiver to assist in supported goal setting’.*
**Benefit Theme 2: Decision support and goal development** This theme highlights the benefits of collaborative goal setting and decision‐making to intervention success.	‘*Goal setting is good. Goals drive motivation to engage. Goals show us the person′s choice and preference. It also needs to be individualised and realistic’*.	**Challenge Theme 2: Cultural, language and regional barriers** This theme covers the challenges faced by people from culturally and linguistically diverse (CALD) backgrounds in accessing services or communicating their vision and hearing issues. It also includes the challenge of ensuring an adequate number of sensory therapists for individuals in regional areas.	‘*May be harder for people from CALD backgrounds to access services and/or communicate any vision or hearing issues—interpreters may be required’*. ‘*Will need to ensure there are adequate sensory therapists available to support people in regional areas too’*.	**Modification Theme 2: Information and resource provision** This theme covers the provision of the right information and resources, at the right time, in the right way, to individuals and their networks. Key elements include: ensuring information is accessible, individualised and timely, inclusive goal‐setting, and dementia decision support tools.	‘*To provide the right information at the right time in a way that best suits the individual person. Not to provide all the information at once’*. ‘*Sharing the training with the support network (e.g., sensory therapist or family) to allow the support network to support them. This will provide continuity in training and use of devices’*.
**Benefit Theme 3: Timely access to care** This theme covers the benefits of direct recommendations and referrals to other services in reducing the time taken for recipients to access essential care. It highlights the potential value of the referral component of the SSI, not only to the intervention recipient but to their support network as well.	‘*Recipients and family members might already be overwhelmed with information and responsibilities. Having a direct recommendation and referral to other services can help reduce the time taken for recipients to access other essential care required’*.	**Challenge Theme 3: Cognitive, dementia and age‐related challenges** This theme includes the difficulties faced by home care recipients living with dementia in completing standard hearing and vision assessments, discussing communication strategies, and participating in goal‐setting activities. It also covers the need for additional training for older individuals with cognitive impairment and their caregivers.	‘*Some home care recipients living with dementia may find it difficult to complete standard hearing and vision assessments*’ ‘*Some people (e.g., living with dementia) may not be able to take part in goal‐setting independently*’.	**Modification Theme 3: Extended training** This theme involves expanding device training to digital and assistive technologies, implementing practical workshops and group discussions for the older adult, family and friends, and providing additional training for caregivers of individuals living with dementia.	‘*Communication training should involve family and friends. In addition to the physical materials, workshops/group discussions involving family and friends are essential because communication requires more than the person with the hearing/vision impairments’*. ‘*It′s not just about the hearing or vision devices or aids but other devices installed. It needs to be holistic for that individual. This ensures that they are able to use these adapted equipment or devices’*.
**Benefit Theme 4: Ongoing support** This theme underscores the crucial role of a multisession intervention design. It emphasises the importance of extending support beyond the initial assessment to include ongoing training opportunities, ensuring skills are reinforced and sustained over time. The use of repeated intervention sessions across an extended period, with repetition of components within this time, was drawn out as being highly valued.	‘*Crucial to have continuous assessment and training. Functioning changes, sometimes rapidly and this is not identified. The continuous assessment ensures continuity of care. Continuous training is also important because some individuals may require repeated instructions and reminders’.* ‘*Devices can take a long time to acclimatise to (e.g., 3‐6 months for hearing aids) so people will need ongoing support as they get used to them’*.	**Challenge Theme 4: Coordination and implementation challenges** This theme covers the challenges posed by the involvement of multiple hearing and vision providers, and the practical implementation of communication strategies by family members and friends.	‘*Multiple hearing and vision providers may make it harder for the sensory therapist to liaise between them all, communicate, and/or to arrange referrals and follow up’*. ‘*Leaflets and other physical materials are fantastic but putting appropriate communication strategies into practice on a daily basis can be challenging, especially for family members/friends that do not having vision/hearing concerns’*.	**Modification Theme 4: Addressing disparities, cultural sensitivity and inclusivity** This theme involves developing strategies to ensure an adequate number of sensory therapists are available in regional areas, as well as measures to address cultural and language barriers, such as the provision of interpreters for effective communication and ensuring cultural inclusivity during home‐based assessments.	‘*Need to ensure there are some CALD options for people too (and/or interpreters available), and perhaps even some picture‐based materials as needed too’*.
**Benefit Theme 5: Effective communication and inclusion** This theme covers the crucial role of effective communication, especially for individuals with hearing and vision issues. It also includes the promotion of hobbies and social inclusion aspects and connecting home care recipients with activities of their preference.	‘*Effective communication is not always discussed. People then take communication for granted. I really appreciate this component because with hearing and vision concerns, communication needs to be adjusted slightly to create the best environment to include the person into conversation’*. ‘*At the end of the day, we want hearing/vision devices to work to ensure that recipients can continue doing what they love and maintain their quality of life. So this is an excellent component of the intervention’*.	**Challenge Theme 5: Sensory therapist challenges** This theme covers the need for sensory therapists to have a comprehensive understanding of different professions and more specialised training to allow the sensory therapist to support individuals with dual sensory loss.	‘*For this to work, the sensory therapist needs to have a good grasp of knowledge about the different professions. To know the scope of practice of each profession and what they could do for the client. Without that knowledge, they would not be able to identify a referral’*.	**Modification Theme 5: Training and development** This theme covers the need for continuous and specialised training for Sensory Therapists. It highlights the importance of ongoing training for the Sensory Therapist regarding the latest technology, interdisciplinary knowledge, limitations of hearing and vision devices, and effective communication strategies for diverse needs.	‘*Ongoing sessions with the recipient, caregiver, and sensory therapist sound great—how will the sensory therapist be able to keep on top of all the different hearing aids and assistive listening devices available? Ongoing training will be important for the sensory therapist’.*

### 3.1 Benefit Themes

Five themes were crafted from the participant responses to the workshop 1 prompt: ‘What do you like about each of the intervention components?’. These themes were: (1) A strong foundation, (2) Decision support and goal development, (3) Timely access to care, (4) Ongoing support, and (5) Effective communication and inclusion. Together, they provide a more consolidated view of the various aspects of hearing and vision support work and its potential benefits.

### 3.2 Challenge Themes

Responses to the prompt ‘what do you dislike about each of the components?’ were analysed, and five themes were derived, which provided a comprehensive overview of the perceived challenges of the intervention and its components. (1) Resistance and denial; (2) Cultural, language and regional barriers; (3) Cognitive, dementia and age‐related challenges; (4) Coordination and implementation challenges; and (5) Sensory therapist challenges.

### 3.3 Modification Themes

To identify themes for modifying intervention components, we included responses to the prompts ‘what components require adjustment or refinement to better meet the identified needs’ as well as the prompt ‘what do you dislike about each of the components?’. The decision to add responses to the prompt regarding dislikes to the modification data was taken as participants often responded to this prompt with action words, like ‘refer’, ‘provide’ and ‘demonstrate’. Excluding this data could result in missing valuable insights.

Five themes relating to areas for modification were uncovered. (1) Tailored services and support; (2) Information and resource provision; (3) Extended training; (4) Addressing disparities, cultural sensitivity and inclusivity; and (5) Training and development.

### 3.4 Suitability Analysis

**Figure 3:**
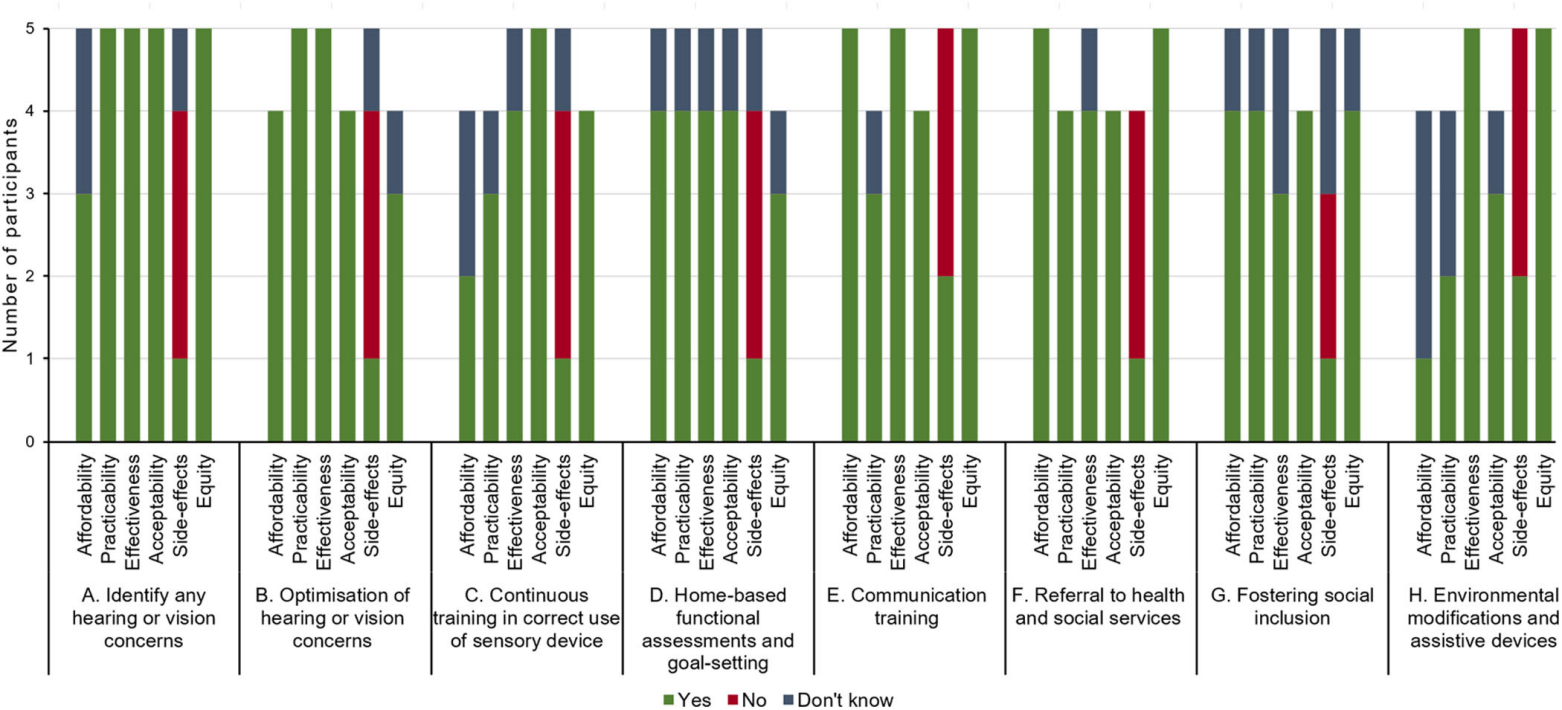
Participant ratings of the perceived affordability, practicability, effectiveness, acceptability, side effects and equity of the SSI by component.

Participant ratings of the APEASE criteria for the various intervention components are shown in Figure 3. All aspects received predominantly positive ratings except for side effects, which are reverse‐scored, with a lower score indicating that fewer negative side effects are expected.

### 3.5 Intervention Development

Appendix A provides an overview of the intervention materials developed or adapted following the co‐design workshops, demonstrating how modification themes were addressed. Themes related to intervention benefits required no changes, while challenges and modification themes were integrated into the intervention. Key examples of the ways the co‐design input shaped the intervention are provided below.

A Sensory Therapist Training Module was added to the intervention in response to the participants' emphasis on the need for strong Sensory Therapist knowledge, as captured in Modification Theme 5. Suggestions made in Workshops 3–5 regarding how best to achieve this were incorporated as part of the development. Examples of these incorporated suggestions included adding information around the functional impact of dual sensory loss and communication training resources. Strategies to address Challenge Theme 1 ‘Resistance and Denial’ were incorporated into the Sensory Therapist Training Module. Healthy ageing researchers also highlighted that the goal‐setting aspect of Component D can identify potential benefits and ‘small wins’ that the program could help achieve and that this is likely to be effective in changing mindset.

The impact of the co‐design process is also apparent in the creation of proforma referrals for the Sensory Therapist to utilise when referring intervention recipients to audiologists or optometrists as part of component A. This resource was created in response to the Challenge Theme 4 ‘Coordination and Implementation Challenges’. The contents of the proforma were shaped by input from the research experts who recommended the inclusion of information such as the participant's lifestyle and goals and information about the intervention.

The Find an Optometrist tool was added to Components A and B to identify dementia‐friendly optometrists, addressing Modification Theme 1 and Challenge Theme 3. This tool will allow the Sensory Therapist to ensure referrals are made to optometrists with appropriate training for this population.

While discussion around Modification Theme 4 ‘Addressing disparities, cultural sensitivity and inclusivity’ led to the conclusion that the issues raised here are predominantly issues of scalability and sustainability, there were still adaptations made to the developed materials to optimise inclusivity. For example, the Functional Assessment Checklist from Component D, utilised in the initial SENSE‐Cog, was modified so that representative images are presented as each activity of daily living is discussed. The use of pictures to support the discussion improves accessibility for people from culturally and linguistically diverse backgrounds [41] as well as for people with dementia [42].

Modification Theme 2 ‘Information and resource provision’ had a significant impact on the way materials were developed across multiple components. A prime example of this was the addition of the Identification of Relevant Communication Tactics tool to Component E. This tool uses a branching scenario to ensure that recipients are presented with communication tactics that are relevant to the challenges they are experiencing, rather than generalised communication tactics.

In response to Modification Theme 3 ‘Extended Device Training’, experts recommended a range of tools that may be beneficial for home care recipients with hearing and/or vision impairment, including speech‐to‐text apps, smartphone accessibility tools, magnifiers, television devices, large button radios and apps such as Be My Eyes. Information regarding these tools was collated and included in Component H.

### 3.6 Usability Data Analysis

Feedback from researchers who participated in Workshops 3, 4 and 5 and the wider research team, regarding the developed intervention materials, was collated and de‐identified. A coding scheme for categorising usability data was adapted from Peute et al. [43], with six of seven codes retained. These were: (1) ease of use, (2) error messages/help instructions, (3) meanings of labels/terminology, (4) layout/screen organisation, (5) graphics/symbols and (6) navigation. Two additional codes, (1) content and (2) grammatical errors, were added after preliminary data examination. One author (H.G.) independently coded the raw data, which was verified by a second author (M.T.).

**Figure 4:**
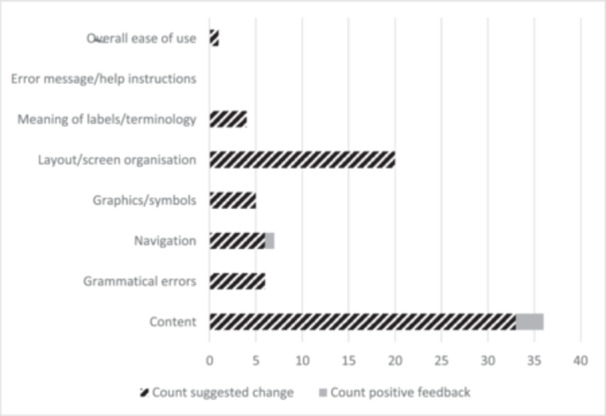
Count of categorised feedback on developed intervention materials provided by researchers following initial development. Feedback was classified by type and as either a requested change or as positive feedback needing no change.

Figure 4 shows the count of the feedback provided by usability code. The largest amount of feedback provided was classified as being related to the content itself (*n* = 36). Examples of comments falling under this classification included: adding information around open captioning services to Component G, expanding the health services discussed in Component F, and clarifying where assistive devices are used with and without hearing aids in Component H.

Issues regarding the layout/screen organisation were the second largest area of revision requested (*n* = 20), with comments such as reordering information in content‐heavy parts of Component C by level of importance, and clearly separating the activity decision aid from the community group information in Component G.

No issues were flagged regarding error messages/help instructions, and minimal feedback was given on ease of use (*n* = 1).

### 3.7 PPI: Workshops 6–8

The GRIPP2‐SF (Guidance for Reporting Involvement of Patients and the Public—Short Form) [44] has been used to structure reporting of the Patient and Public Involvement (PPI) activities in this study. Use of this standardised form of reporting has been promoted in the literature as a way of ensuring consistency and transparency [45].

#### 3.7.1 Aims

PPI was incorporated to gain further feedback regarding the usability of the intervention materials for the target population and to enhance the patient‐centredness of the intervention. Previous research has found that consumer involvement has led to more patient‐centred and effective health interventions [46].

#### 3.7.2 Methods

Three workshops were undertaken with three PPI members. Participants in these workshops were aged 65 years or older and were family members of older adults living with dementia and sensory impairments who had received home care. Due to the age of the PPI members, it was possible that they had a sensory impairment themselves, but this was not a requirement of participation.

The first workshop oriented them to the purpose of the intervention and the materials themselves (*engage & plan*). Members then reviewed the Canvas‐based materials in their own homes. They were welcome to share the materials with their family members living with dementia if they wished to, but this was not explicitly required. In the second workshop, participants discussed their experiences with the intervention materials using a retrospective think‐aloud approach, sharing their insights and difficulties. A third workshop presented the participants with a revised version of the intervention materials, and the participants were encouraged to provide any additional feedback and insights regarding the suitability of the changes made in response to their feedback.

#### 3.7.3 Results

Patient and public feedback was transcribed and analysed using the coding method outlined in the usability data analysis section. Initial coding of Workshop 2 was completed by M.T. and independently reviewed by H.G., whereas data from PPI Workshop 3 was initially coded by H.G. and independently reviewed by M.T. Appendix B shows the feedback count by usability code for Workshops 2 and 3.

In Workshop 2, the highest amount of feedback provided concerned the choice of graphics and symbols (*n* = 28), followed by layout/screen organisation (*n* = 23). Issues flagged included feeling the images used were not relatable or did not reflect the Australian environment, and the use of colour schemes that they felt would be difficult for people with vision impairment. Interestingly, this occurred despite the use of the WCAG Color Contrast Checker tool (https://accessibleweb.com/color-contrast-checker/) during intervention material development. Consistency in layout choices was raised in the layout/screen organisation category, with members flagging issues such as text justification not being consistently left‐aligned across pages.

Fewer changes were requested at the third workshop (*n* = 107 versus *n* = 30). Layout/screen organisation reduced but remained the highest category of feedback (*n* = 11). Requested changes included an increase in spacing on pages to improve readability. Feedback regarding terminology increased (*n* = 4 versus *n* = 7), with suggestions to make terminology more relatable. For example, participants suggested renaming Component G from ‘Fostering Social Inclusion’ to include the word Connecting. This was implemented, and the component was relabelled as ‘Connecting with Others and Interests’. The materials were also reviewed for suitability at a year 8 literacy level.

#### 3.7.4 Outcomes

Feedback provided by the PPI members was used to guide modifications to the intervention. These alterations included changes in graphics, layout, terminology, readability and relatability.

#### 3.7.5 Critical Perspective

The chosen method of engaging with members of the public was highly effective in identifying areas of the intervention which needed improvement that were not previously picked up by other stakeholders. This highlights the unique perspectives that members of the public bring to intervention development.

## 4 Discussion

This study utilised a co‐design approach to tailor the SENSE‐Cog intervention for Australian older adults with hearing and/or vision impairments in home care settings, including those with cognitive impairment. By employing elements of Boyd's co‐design model [31], we engaged with relevant professionals across a series of five workshops and gained insight into the adaptations needed to optimise the SENSE‐Cog intervention for the Australian home‐care context. The findings highlighted the multifaceted nature of the potential benefits and challenges associated with the delivery of a hearing and vision intervention to a cognitively diverse population.

The co‐design process led to modifications to each component of the intervention as well as to the creation of a new training module for the Sensory Therapist who will administer the intervention to ensure adequate knowledge to deliver the intervention to its fullest potential. The adapted Australian SENSE‐Cog intervention places an increased focus on providing resources for various levels of cognitive ability, making resources accessible outside of intervention sessions via online materials, and tailoring training provided during the intervention to meet the individual's goals. The tailoring of training to the individual's goals is consistent with the recommendations of Skivington et al. [26].

The adaptation of the intervention materials to online resources is another point of difference from the original SENSE‐Cog intervention. The inclusion of online resources increases the accessibility of the materials not only to the home care recipient during intervention sessions but also to their family members and caregivers, which was highlighted as important during the co‐design process. As of 2021, 93% of older adults in Australia reported having internet access in their homes, making online resources a useful way to access intervention materials for the target population [47]. Furthermore, the inclusion of online resources in existing hearing interventions has been shown to lead to improved hearing aid skills and outcomes [48].

Interestingly, the increased focus on information provision to care partners is consistent with an adaptation need identified in a study by Sheikh et al. [49] who adapted the SENSE‐Cog intervention for South Asian settings. The need for information provision to care partners may not necessarily be a culturally specific need, but an area which could be strengthened in the intervention in general. In contrast to the Australian adaptation, the South Asian adaptation of SENSE‐Cog led to the *removal* of the component focusing on social inclusion, which was flagged as being valuable in the Australian study. This difference in adaptations highlights the need to avoid generalising across contexts.

The co‐design process reported here has several strengths. The use of an iterative analytical approach ensured that a deep understanding of the data was gained. Morgan and Nica highlighted that iterative analysis can lead to more robust and meaningful results [50]. Involvement of three researchers also provided multiple points of view in the analysis and likely minimised bias, strengthening the validity of the findings. The use of a standardised co‐design model to guide the process further strengthens the scientific validity of the approach.

Public and patient involvement has previously been shown to have a considerable impact on research with people with dementia [51], and PPI in the adaptation of the SENSE‐Cog intervention for the Australian home care setting led to meaningful changes in the intervention. This change in usability aligns with Carman et al., who emphasise that patient involvement at the development stage should improve acceptability [52]. It is interesting to note that the feedback provided by the members of the public and patient involvement group differed from that provided by research experts; members of the public were focused on usability, while the researchers focused on content. This point of difference highlights the value of engaging with the public and patients to obtain views that differ from those of subject experts.

There are limitations to the current work. Workshop group sizes for hearing and vision professionals were small due to low recruitment responses, leading to the possibility that views gained may not fully reflect the larger population of professionals. Future research could examine the generalisability of the themes identified in the current work. Additionally, as the workshop participants were given the option of sharing their thoughts anonymously via Padlet, it was not possible to attribute the comments to the individual participant or to reflect on how their specific expertise may have shaped their input. This decision was made to ensure participants felt comfortable contributing in a group setting.

The adapted intervention has implications for home care providers, caregivers and policymakers. By addressing the hearing and vision needs of older adults, home‐delivered interventions may enhance quality of life, promote independence and foster social engagement. The process of co‐design with audiologists, optometrists, home care staff and research experts led to changes in the SENSE‐Cog intervention to improve its applicability to the Australian home care setting. A field trial of the intervention is planned to determine its efficacy and cost‐effectiveness. Future research should also explore the long‐term impacts of sensory support interventions, including quality of life, healthcare utilisation, caregiver relationship satisfaction and social engagement.

If found to be cost‐effective, policymakers should consider providing funding and resources to support the implementation and scaling of intervention in home care settings, as this will ensure that older adults with hearing or vision impairments receive the necessary support to maintain their independence and quality of life.

## Author Contributions

**Helen Gurteen:** writing – original draft, methodology, investigation, data curation, formal analysis, validation. **Melinda Toomey:** investigation, methodology, formal analysis, data curation, writing – review and editing, validation. **Bronwyn Franco:** formal analysis, writing – review and editing, validation. **Najwan El‐Saifi:** writing – review and editing, methodology. **Chyrisse Heine:** methodology, funding acquisition, writing – review and editing. **Iracema Leroi:** conceptualisation, funding acquisition, writing – review and editing, methodology. **Carly J. Meyer:** funding acquisition, methodology, writing – review and editing. **Sheela Kumaran:** funding acquisition, methodology, writing – review and editing. **Piers Dawes:** conceptualization, supervision, methodology, funding acquisition, writing – review and editing.

## Disclosure

The funder had no role in the design or reporting of this research study.

## Ethics Statement

This study was reviewed and approved by The University of Queensland Human Research Ethics Committee, approval number 2023/HE001081.

## Consent

Written consent was obtained from participants.

## Conflicts of Interest

The authors declare no conflicts of interest.

## Data Availability

The de‐identified datasets generated during this study will be made publicly available via The University of Queensland Research Data Manager (UQ RDM).

## Appendix A Summary of intervention materials developed or refined, cross‐referenced with the modification theme addressed

**Table:**

Component	Aim	Co‐design modification theme addressed	Elements included or developed
Sensory Therapist Training Module	To provide the sensory therapist with the necessary knowledge to deliver the SSI to people with diverse needs.	Modification Theme 5: Training and Development	– Introduction to the SSI – Dementia training – Hearing and Hearing Loss – Vision and Vision Loss – Dual Sensory Loss – Allied Health Professionals – Overview of the SSI – Communication Skills Training – Behaviour Change and Adherence Promotion – Supporting Decision‐Making – Home Care
A. Identify Sensory Concerns	To provide useful resources on hearing and vision loss and how to access care	Modification Theme 1: Tailored Services and Support Modification Theme 2: Information and Resource Provision Modification Theme 4: Addressing Disparities, Cultural Sensitivities and Inclusivity	– Personal Sensory Plan Questionnaire – Referral proformas for referral to audiologists or optometrists – Hearing Hub – Hearing Loss – Hearing Aids – Hearing Aid Features – Tinnitus – Vision Hub – Vision Loss – Refractive Errors – Lens Types – Lens Materials – Practitioner Hub – Audiologists and The Hearing Assessment – Find an Audiologist – Optometrists and the Eye Exam – Find an Optometrist
B. Optimising Hearing and Vision Health	To provide helpful resources on how to access hearing and vision care	Modification Theme 1: Tailored Services and Support Modification Theme 2: Information and Resource Provision	– Hearing Loss Information – Tinnitus – Audiologists and the Hearing Test – Preparing for my Hearing Test – Find an Audiologist – Optometrists and the Eye Exam – Preparing for my Eye Exam – Find an Optometrist
C. Sensory Device Training	To provide resources on correctly using and maintaining hearing and vision devices	Modification Theme 1: Tailored Services and Support Modification Theme 2: Information and Resource Provision Modification Theme 3: Extended Training	– Hearing Aid Skills and Knowledge (HASK) Tool – Rechargeable Hearing Aid Skills and Knowledge (R‐HASK) Tool – Hearing Aid User Guide – Glasses and Vision Skills and Knowledge (GLASK) – Glasses User Guide
D. Home‐Based Functional Assessment	To support activities of daily life that are impacted by hearing or vision, and to set goals around what the home care recipient wishes to achieve from the SSI.	Theme 1: Tailored Services and Support Modification Theme 2: Information and Resource Provision Modification Theme 3: Extended Training	– Bangor Goal Setting Inventory (Short Version) – Functional Assessment Checklist
E. Communication Training	To provide guidance for effective communication when living with hearing, vision and cognitive impairment	Modification Theme 1: Tailored Services and Support Modification Theme 2: Information and Resource Provision Modification Theme 3: Extended Training	– Identifying Relevant Tactics – 1:1 Communication Training – Group Communication Training – Written Communication Training – Communication on the Phone – Communication Tips for Others
F. Health and Social Services Support	To provide recommendations for referral to health and social services as related to hearing and vision needs.	Theme 1: Tailored Services and Support Modification Theme 2: Information and Resource Provision Modification Theme 3: Extended Training	– Health Services – Social Services – Hearing and Vision Advocacy – Healthy Ageing Advocacy
G. Fostering Social Inclusion	To provide guidance on connecting with your community, social activities and hobbies	Theme 1: Tailored Services and Support Modification Theme 2: Information and Resource Provision Modification Theme 3: Extended Training	– Hobby/Activity Decision Aid – Community Groups – Hearing and Vision Support Groups – Audio Description and Subtitling
H. Environmental Modifications and Assistive Devices	To provide valuable information on home‐based environmental modifications and assistive devices that can support vision and hearing needs	Theme 1: Tailored Services and Support Modification Theme 2: Information and Resource Provision Modification Theme 3: Extended Training	– Auditory Environment Modifications – Visual Environment Modifications – Dementia‐friendly Environment – Assistive Technologies

## Appendix B PPI Workshop Usability Analysis Results. Feedback was classified by type and as either a requested change or a positive feedback needing no change

.
